# 

**Published:** 1848-02

**Authors:** 


### CONTENTS.

### PART I.—ORIGINAL COMMUNICATIONS.

ART. 1. *Cases and observations on the use of Strychnia in Intermittent Fevers.* By J. E. McGirr, A. M., M. D., Chicago.

ART. 2. *Letter* from S. G. Armor, M. D.

ART. 3. *Letter* from Dr. Welch.

ART. 4. *Remarks in relation to the Pathological Anatomy of the mucous membrane of the intestinal canal during infancy.* By Drs. Friedleben and Flesch, of Frankfort on the Main.

ART. 5. Dr. Bird's *Report of the Cook County Hospital.*

ART. 6. *Aneurismal Varix of the popliteal space—ligature of the artery and vein. Hemorrhage, Amputation, recovery.* By D. Brainard, M.D.

### PART II.—REVIEWS.

ART. 7. *Physiological Anatomy and Physiology of Man, with numerous original illustrations.* By Drs. Todd and Bowman.

### PART III.—BIBLIOGRAPHICAL NOTICES.

ART. 8. *The Half Yearly Abstract of Medical Sciences.* Edited by W. H. Rankin, M. D.

ART. 9. *The Principles and Practice of Midwifery.* By David H. Tucker, M. D.

ART. 10. *Twenty-seventh Annual Report of the Bloomingdale Asylum for the Insane.* By Pliny Earle, M. D.

*Fifth Annual Report of the Managers of the* (*N. Y.*) *State Lunatic Asylum, made to the Legislature, Jan.* 1848.

*Report of the Pennsylvania Hospital for the Insane for the year* 1847. By Thomas S. Kirkbride, M. D.

### PART IV.—SELECTIONS.

1. On a new criterion for the regulation of Blood-letting.
2. On the treatment of Fever by Cold Water.
3. On the employment of Inhalation of Ether in some forms of Ophthalmia.
4. On the use of Ocular Douches for the treatment of Purulent Ophthalmia of infants, Opacities of the Cornea, &c.
5. A case of rupture of the Womb occurring during labor, and followed by recovery.
6. On the source and influence of Malaria in the South-west.
7. On the antagonism between Typhoid Fevers, Intermittents and Phthisis.
8. On the frequent occurrence of Alkaline Urine in Health, &c.
9. Cause of Mortality in Still-born Children.
10. Abortion, and Menstruation during Pregnancy.
11. The case of Mr. Whitman.
12. The Bite of the Rattlesnake.
13. Nitrate of Silver in Membranous Croup.
14. Second Advent of Thomsonism.
15. Surgical Co-apter and Splints.
16. Annual Meeting of Wisconsin Medical Society.
17. Abdominal Tumor mistaken for Pregnancy.

### PART V.—EDITORIAL.

ART. 1. Our Journal.

ART. 2. Indianapolis Medical Society.

ART. 3. Chloroform—the new Anæsthethic Agent.

